# Clinical application of the F21 multipurpose cystoscope with continuous irrigation capability

**DOI:** 10.3389/fsurg.2026.1680966

**Published:** 2026-03-05

**Authors:** Guihua Cao, Tao Ma, Liangcheng Liu, Wei Li, Qiang Li, Jianping Du

**Affiliations:** Department of Urology, The People's Hospital of Leshan, Leshan, China

**Keywords:** benign prostatic hyperplasia, continuous irrigation, cystoscope, F21 multipurpose cystoscope, GreenLight laser vaporization of the prostate

## Abstract

**Objective:**

To evaluate the safety and clinical performance of an F21 multipurpose cystoscope equipped with a continuous irrigation system in routine urologic procedures.

**Methods:**

This single-center retrospective study consecutively enrolled 150 patients undergoing F21 multipurpose cystoscope–assisted procedures (50 double-J stent removals, 50 retrograde 5-Fr ureteral catheterizations, and 50 BPH patients receiving PVP).BPH patients had bladder outlet obstruction (Qmax <10 mL/s), and major exclusions included prostate cancer and prostate volume >80 mL.Outcomes included procedure-related complications; IPSS and Qmax were assessed preoperatively and at 3 and 6 months in the PVP cohort.

**Results:**

All double-J stent removals and retrograde catheterizations were completed successfully under local anesthesia with a consistently clear operative field, and no postoperative complications were observed. All 50 PVP procedures were completed successfully; mean operative time was 65.1 ± 16.9 min, blood loss was <60 mL, postoperative irrigation averaged 17 h, and catheter removal occurred on postoperative days 3–5. In the PVP group, Qmax increased from 6.314 preoperatively to 21.716 at 3 months and 21.006 at 6 months, while IPSS decreased from 23.540 to 4.700 and 4.420, respectively (all *p* < 0.001). At the 3-month and 6-month postoperative follow-up time points, none of the patients developed complications such as urinary incontinence, urethral stricture, bladder neck contracture, or voiding dysfunction.

**Conclusion:**

The F21 multipurpose cystoscope with continuous irrigation provides stable visualization and supports both routine cystoscopic interventions and PVP in a small-caliber platform, demonstrating favorable safety and functional outcomes.

## 1 Introduction

Cystoscopy is one of the most commonly used diagnostic tools in urology. Similar to gastroscopy and colonoscopy, it belongs to the category of endoscopic instruments (1). Clinically, it is frequently employed for procedures such as cystoscopic examination, biopsy, foreign body removal, retrograde catheterization, and ureteral stent placement (2). Conventional cystoscopes typically provide only intermittent bladder irrigation, requiring repeated filling and drainage to maintain visualization, which can interrupt workflow and lead to an unstable operative field (3). Although resectoscopes offer continuous inflow–outflow, their larger caliber (e.g., F24/F26) may be unsuitable for patients with a narrow urethra or concomitant urethral stricture and may increase the risk of urethral mucosal injury (4). Therefore, a practical gap remains for a small-caliber platform that can provide continuous irrigation while preserving routine cystoscopic functions and supporting laser-based procedures. To address this limitation, we developed the F21 multipurpose cystoscope capable of continuous irrigation through a collaborative effort between clinicians and engineers (Supplementary File 1, Utility Model Patent No: 202323218027.1). The F21 multipurpose cystoscope enables sustained bladder irrigation during cystoscopy, thereby ensuring procedural continuity. In addition to the standard functions of a conventional cystoscope, it is also applicable to transurethral laser vaporization procedures for benign prostatic hyperplasia (BPH). It is particularly advantageous for patients with urethral strictures who are unsuitable for conventional F24/F26 resectoscope. This novel approach has demonstrated satisfactory clinical outcomes, as detailed in the following report.

## 2 Study design

This study was a single-center, retrospective clinical application research conducted in the Department of Urology, Leshan People's Hospital. The primary objective was to evaluate the safety and feasibility of a novel F21 multi-purpose cystoscope with continuous irrigation function in routine urological procedures.

Since this study did not involve random grouping, interventional group comparison, or experimental treatment protocols, it was not prospectively registered on any public clinical trial registration platform. The study protocol had been reviewed and approved by the Ethics Committee of Leshan People's Hospital [Approval No.: LYLL (2024) KY 137]. The entire research process was carried out in strict compliance with the Declaration of Helsinki and relevant local regulations. Written informed consent was obtained from all participants prior to their enrollment in the study.

A consecutive enrollment method was adopted, and patients who received treatment in our department during the study period were recruited. To minimize selection bias, all eligible patients who met the predefined inclusion criteria and had no exclusion criteria were invited to participate. According to the clinical indications, the enrolled patients were divided into three groups, who respectively underwent F21 multipurpose cystoscope double-J stent removal, retrograde ureteral catheterization, and PVP for the treatment of BPH.

## 3 Clinical data

### 3.1 Inclusion criteria and sample size

**Figure 1:**
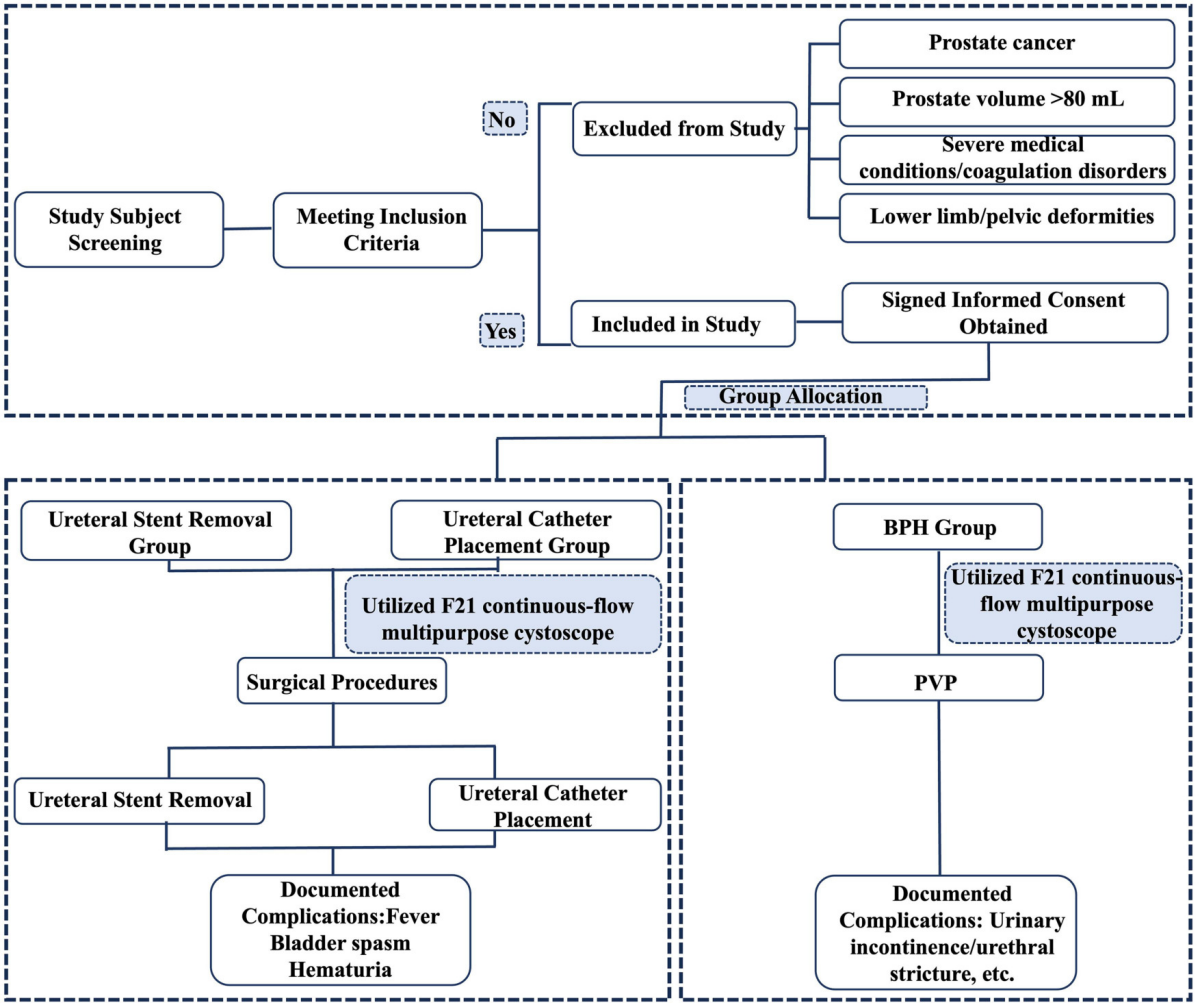
Flowchart diagram illustrating study subject screening, inclusion and exclusion criteria, group allocation,and subsequent clinical procedures, with documented complications for ureteral stent removal, ureteral catheter placement, and BPH groups.

A total of 150 patients were enrolled, including 50 patients undergoing routine local anesthesia for double-J stent removal, 50 patients receiving retrograde placement of an F5 ureteral catheter, and 50 patients diagnosed with BPH (Figure 1). Urodynamic studies in the BPH group indicated bladder outlet obstruction with a maximum urinary flow rate (Qmax) of less than 10 mL/s.

### 3.2 Exclusion criteria

Patients were excluded if they had a diagnosis of prostate cancer, a prostate volume greater than 80 mL, severe coagulopathy, or congenital deformities of the lower limbs or pelvis. All participants were informed in detail about the surgical procedure and potential risks, and signed a written informed consent form before the operation.

### 3.3 Patient characteristics (BPH group)

A total of 50 patients with BPH were included, aged 58–85 years, with a mean age of 71.5 ± 8.1 years. All presented with lower urinary tract obstruction symptoms, and 5 had indwelling catheters due to urinary retention. Transrectal ultrasonography showed prostate volumes ranging from 30 to 80 mL, with an average of 53.4 ± 15.8 mL. Post-void residual urine volume ranged from 60 to 380 mL, averaging 143.5 ± 70.5 mL. Qmax ranged from 3 to 10 mL/s. Prostate-specific antigen (PSA) levels were all below 4 ng/mL. The preoperative International Prostate Symptom Score (IPSS) ranged from 15 to 30. None of the patients had a history of urethral trauma or prior urethral surgery. Comorbidities included hypertension in 5 cases and diabetes mellitus in 3 cases. Routine preoperative evaluations, including coagulation profile, serum biochemistry, electrocardiogram, and chest radiography, revealed no contraindications to surgery. Five patients had urinary tract infections, and surgery was performed after urine cultures and antibiotic sensitivity testing guided appropriate antimicrobial therapy and infection resolution.

## 4 Methods

### 4.1 Fabrication of a multipurpose cystoscopic working inner sheath with continuous irrigation capability

**Figure 2:**
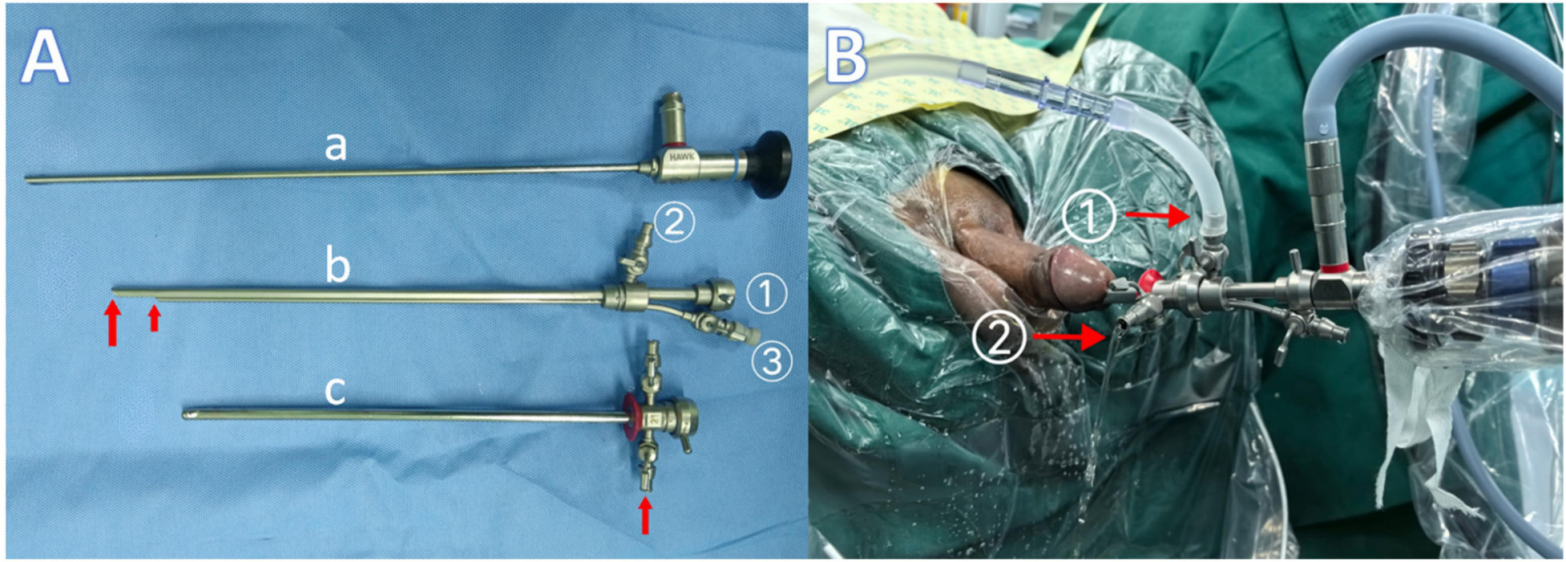
**(A)** Endoscope; b: Photograph of the working inner sheath, with the following labeled components: (1) Entry port for the endoscope; (2) Inlet for the irrigation channel; (3) Entry port for the working (instrument) channel; c: Outer sheath of the F21 cystoscope, indicating the outlet of the outflow channel (as shown by the arrow). **(B)** Arrow (1) Inlet of the irrigation channel located on the working inner sheath; Arrow (2) Outlet of the outflow channel located on the outer sheath of the cystoscope.

Based on the standard F21 cystoscope (Hawk, China), the F21 multipurpose cystoscope was designed and fabricated to enhance operative efficiency. Constructed from medical-grade stainless steel, the F21 multipurpose cystoscope incorporates three primary channels: an endoscopic channel, an irrigation (inflow) channel, and an instrument (operating) channel (Figure 2A). The distal end of the inflow channel features two outflow openings to ensure adequate irrigation volume. The space between this newly developed inner sheath and the outer sheath of the F21 cystoscope serve as the outflow (drainage) channel. Irrigation fluid exits from the bladder through the outer sheath's drainage port. This design allows for continuous bladder irrigation during various surgical procedures while maintaining a clear operative field (Figure 2B). The stainless steel inner sheath is reusable and can be sterilized using low-temperature plasma disinfection.

### 4.2 Surgical method

#### 4.2.1 F21 multipurpose cystoscope removal of double-J stent and retrograde placement of F5 ureteral catheter

**Figure 3:**
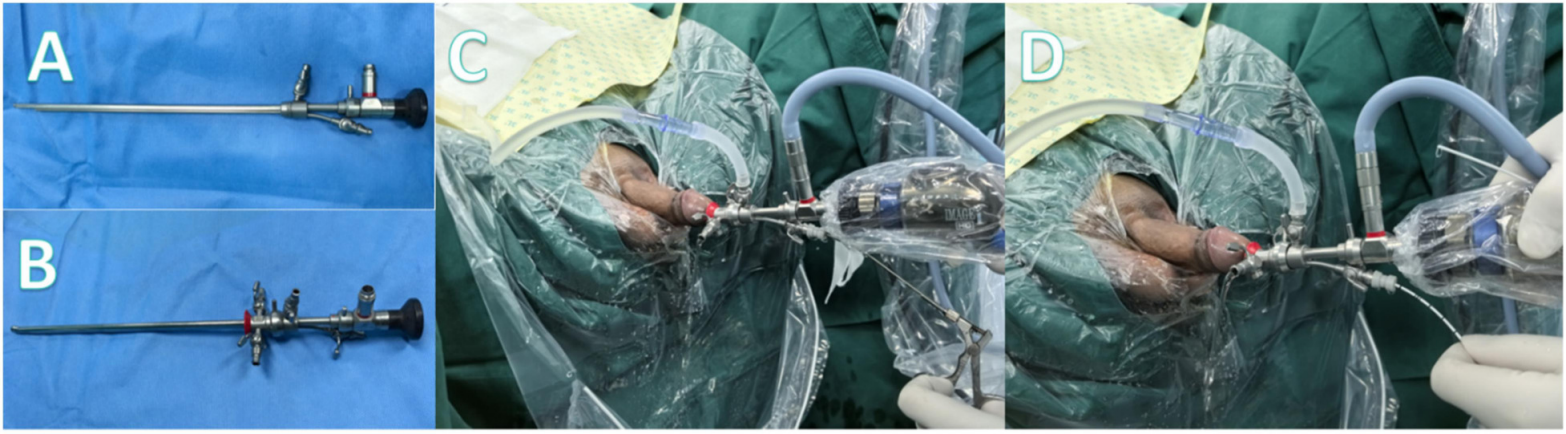
**(A)** Insertion of the endoscope into the working inner sheath; **(B)** Insertion of the inner sheath into the outer sheath of the F21 cystoscope; **(C)** Extraction of the double-J stent; **(D)** Retrograde insertion of the ureteral catheter.

With the patient in the lithotomy position, routine disinfection and draping were performed. Local anesthesia was carried out by using lidocaine gel with a concentration of 2 percent. A 30° Hawk endoscope was first inserted into the novel working inner sheath (Figure 3A), which was then inserted into the F21 outer sheath of cystoscope (Figure 3B). Under direct vision, the assembled cystoscope was inserted transurethral into the bladder. Either double-J stent removal (Figure 3C) or retrograde placement of an F5 ureteral catheter was performed as required (Figure 3D). Postoperative complications—including fever, bladder spasms, severe hematuria, bladder perforation, and urethral injury were recorded.

#### 4.2.2 PVP

**Figure 4:**
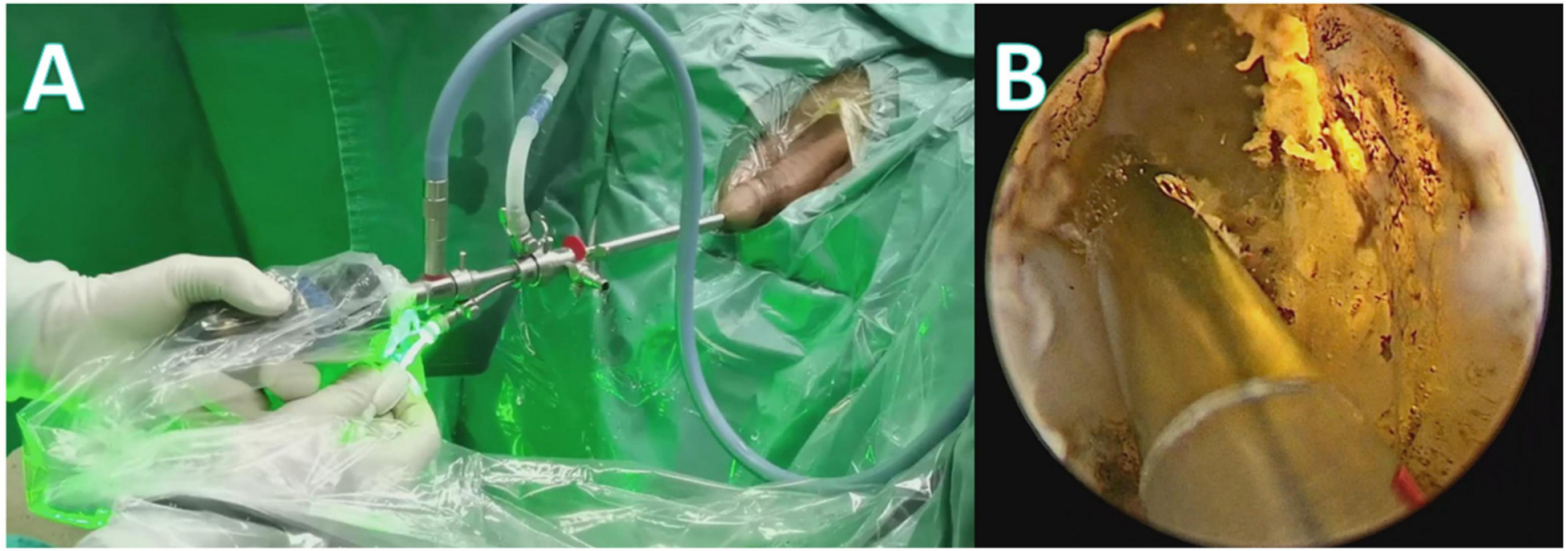
**(A)** External view of the PVP surgery; **(B)** Intraoperative view of the PVP procedure.

Under epidural anesthesia and in the lithotomy position, routine disinfection and draping were performed. The F21 multipurpose cystoscope assembly and insertion procedures were identical to those described previously. The urethra, prostate, and bladder were carefully inspected. A GreenLight laser fiber (Boston Scientific, USA) was then inserted through the working channel of the inner sheath. Following the standard PVP protocol, the median lobe was vaporized from the bladder neck to the level of the verumontanum. Subsequently, both lateral lobes were vaporized, aiming to ablate the hyperplastic prostatic tissue up to the surgical capsule as thoroughly as possible (Figures 4A,B). At the end of the procedure, a 20F three-way Foley catheter was inserted, and continuous bladder irrigation with normal saline was initiated. Irrigation was discontinued once the effluent became clear. Postoperative follow-up was conducted in the outpatient clinic at regular intervals, documenting changes in the IPSS, Qmax, and any complications.

## 5 Results

### 5.1 F21 multipurpose cystoscope removal of double-J stent and retrograde placement of F5 ureteral catheter

All procedures for double-J stent removal (*n* = 50) and retrograde placement of 5 Fr ureteral catheters (*n* = 50) were completed under local anesthesia. The surgical field remained clear throughout, with no evidence of bladder overdistension or underfilling. No postoperative complications such as fever, severe hematuria, bladder perforation, or urethral injury were observed.

### 5.2 PVP

All 50 cases of PVP procedures were completed. The operative time ranged from 35 to 90 min, with a mean duration of 65.1 ± 16.9 min. Intraoperative blood loss was less than 60 mL. Postoperative bladder irrigation was maintained for 10–24 h, with an average duration of 17 h. The urinary catheter was removed between postoperative days 3 and 5. Both Qmax and IPSS in the PVP group improved significantly during follow-up (Table 1). Qmax increased markedly from 6.314 preoperatively to 21.716 at 3 months and 21.006 at 6 months postoperatively (mean differences vs. baseline: −15.402 and −14.692, respectively; both *p* < 0.001; Cohen's *d* ≈ 4.0–4.2), indicating a substantial improvement in urinary flow. No significant difference was observed between 3 and 6 months (*p* = 0.333; *d* = 0.194), suggesting that Qmax stabilized after 3 months. Similarly, IPSS decreased from 23.540 preoperatively to 4.700 at 3 months and 4.420 at 6 months (mean differences vs. baseline: 18.840 and 19.120, respectively; both *p* < 0.001; Cohen's *d* ≈ 7.1–7.2), reflecting marked alleviation of lower urinary tract symptoms. The difference between 3 and 6 months was not significant (*p* = 0.596; *d* = 0.106). All 50 patients were followed up at 3 and 6 months postoperatively, and none developed urinary incontinence, urethral stricture, bladder neck contracture, or voiding dysfunction.

**Table 1:** Follow-up outcomes for Qmax and IPSS.

Outcome	(I) Time	(J) Time	Mean (I)	Mean (J)	Diff (I-J)	*p*	Cohen's *d*
	Pre-op	Post-op 3 mo	6.314	21.716	−15.402	0.000***	−4.216
Qmax	Pre-op	Post-op 6 mo	6.314	21.006	−14.692	0.000***	−4.022
	Post-op 3 mo	Post-op 6 mo	21.716	21.006	0.710	0.333	0.194
	Pre-op	Post-op 3 mo	23.540	4.700	18.840	0.000***	7.141
IPSS	Pre-op	Post-op 6 mo	23.540	4.420	19.120	0.000***	7.247
	Post-op 3 mo	Post-op 6 mo	4.700	4.420	0.280	0.596	0.106

***p < 0.01.

## 6 Discussion

The objective of this study was to evaluate the safety and clinical performance of an F21 multipurpose cystoscope equipped with a continuous irrigation system in routine urologic procedures. In our single-center retrospective series, the device enabled stable visualization and successful completion of double-J stent removal, retrograde 5-Fr ureteral catheterization, and PVP, with no procedure-related postoperative complications observed. In the PVP cohort, functional outcomes (IPSS and Qmax) improved significantly at 3 and 6 months, and no major late complications were detected during follow-up.

Since its invention, the cystoscope has evolved from a rigid instrument to a flexible fiberoptic and electronic versions (5, 6), and it remains one of the most widely used tools in urologic practice. Despite these technological advances, conventional cystoscopes still rely on intermittent bladder irrigation and do not provide continuous inflow and outflow in the same manner as a resectoscope. Repeated filling and drainage of the bladder may interrupt the operative rhythm and can make it challenging to maintain a stable visual field, particularly during prolonged or technically demanding maneuvers. The development of an F21 multipurpose cystoscope configuration with continuous irrigation addresses this practical limitation by enabling uninterrupted fluid circulation, thereby maintaining a consistently clear operative field. In our clinical use, this feature improved the procedural experience and supported efficient performance of double-J stent removal, retrograde ureteral catheterization, and PVP.

Compared with resectoscopes, the F21 continuous-irrigation multipurpose cystoscope offers several practical advantages. Resectoscopes are commonly used for transurethral resection of the prostate (TURP); however, their larger caliber and higher invasiveness may increase the risk of urethral mucosal injury, particularly in patients with urethral stricture or other anatomical constraints (7). In contrast, the smaller diameter of the F21 system, combined with continuous irrigation, provides a less invasive option for patients who are not suitable candidates for conventional F24/F26 resectoscope procedures. This technical advantage is especially relevant for patients with BPH complicated by urethral stricture.

In our earlier work, we used a 3D-printed laser working inner sheath to achieve continuous bladder irrigation when paired with an F21 cystoscope, and applied the system to procedures such as PVP and bladder tumor resection with favorable outcomes. However, this early design had notable limitations: it was not compatible with routine cystoscopic functions (e.g., biopsy, stent removal, or catheter placement); the small inflow port required an infusion pump, introducing potential safety concerns; the connection points lacked sufficient mechanical stability; and the titanium alloy material was expensive and difficult to manufacture, limiting scalability and broad adoption (8, 9).

We therefore redesigned the inner sheath to overcome these constraints and established an improved the F21 multipurpose cystoscope assembly that supports continuous saline irrigation while enhancing safety and versatility. The redesigned sheath incorporates a dedicated inflow channel with dual distal ports, while the space between the inner sheath and the standard outer sheath serves as the outflow pathway, enabling balanced, continuous circulation without the need for an infusion pump. Importantly, the working channel accommodates not only laser fibers but also standard cystoscopic accessories (e.g., biopsy forceps, foreign-body forceps, ureteral catheters, and stents), thereby preserving the full functional scope of conventional cystoscopy. The sheath is manufactured from medical-grade stainless steel, permitting sterilization and reuse, while also providing a mature production pathway suitable for broader clinical dissemination.

In this study, F21 multipurpose cystoscope provided a consistently clear operative field during double-J stent removal and retrograde ureteral catheterization, without excessive bladder distension or underfilling. We also confirmed the feasibility and safety of this configuration for PVP. Historically, BPH patients with concomitant urethral stricture who could not undergo conventional resectoscopic surgery often required alternatives such as urethral dilation or suprapubic cystostomy (10, 11). At present, our method offers a promising alternative for such cases, potentially preventing recurrent urethral strictures and reducing the physical and economic burden associated with repeated interventions.

Beyond routine cystoscopic inspection, biopsy, foreign-body retrieval, and retrograde catheterization, the continuous-irrigation F21 platform is compatible with transurethral PVP and could be extended to other laser-based procedures, including laser vaporization techniques for BPH, laser ablation of bladder tumors, and laser lithotripsy for bladder stones. Overall, this innovation expands the functional scope of conventional cystoscopy and shows promising clinical applicability.

Several limitations should be acknowledged. This was a single-center, retrospective clinical application study, and functional outcomes after PVP were assessed over a relatively short follow-up period. Prospective, multicenter studies with longer follow-up are warranted to further define optimal patient selection, evaluate durability of outcomes, and clarify comparative performance vs. established endoscopic platforms.

In conclusion, the F21 multipurpose cystoscope with continuous irrigation provides clear, uninterrupted visualization while maintaining the versatility of conventional cystoscopy in a smaller-caliber system. These features may improve procedural efficiency and safety and offer a useful alternative for patients with anatomical challenges that preclude the use of conventional resectoscopic equipment.

## 7 Conclusion

The novel multipurpose cystoscopic inner sheath enables continuous bladder irrigation, maintaining a clear surgical field. In addition to the capabilities of a conventional cystoscope, it supports transurethral GreenLight laser vaporization of the prostate, offering a safe and effective tool for urological procedures. Future prospective, multicenter studies with larger samples and longer follow-up are warranted to better define optimal patient selection, confirm the durability of outcomes, and clarify comparative performance vs. established endoscopic platforms.

## Data Availability

The datasets presented in this study can be found in online repositories. The names of the repository/repositories and accession number(s) can be found in the article/Supplementary Material.
