# Supplementary material for: Clinical application of the F21 multipurpose cystoscope with continuous irrigation capability
Source: Front Surg. 2026 Mar 5;13:1680966. doi: 10.3389/fsurg.2026.1680966 (PMC12999935; doi:10.3389/fsurg.2026.1680966)
Supplement: Supplementary file 1 [file Datasheet1.pdf]

证书号第21705311号

专利公告信息

实用新型专利证书

实用新型名称：一种循环式多用途膀胱镜鞘结构

专 利 权 人：乐山市人民医院

地　　　址：614000 四川省乐山市市中区白塔街238号

发　明　人：曹贵华;俞颖超

专　利　号：ZL 2023 2 3218027.1　　　授权公告号：CN 221711871 U

专利申请日：2023年11月28日　　　授权公告日：2024年09月17日

申请日时申请人：杭州好克光电仪器有限公司

申请日时发明人：曹贵华;俞颖超

国家知识产权局依照中华人民共和国专利法进行审查，决定授予专利权，并予以公告。

专利权自授权公告之日起生效。专利权有效性及专利权人变更等法律信息以专利登记簿记载为准。

局长

申长雨

国家知识产权局

2024年09月17日
